# Use of the triple coaxial (triaxial) microcatheter system in superselective arterial embolisation for complex interventional cases: an initial experience with the system

**DOI:** 10.1186/s42155-022-00340-z

**Published:** 2022-12-23

**Authors:** Sonam Tashi, Zehao Tan, Apoorva Gogna

**Affiliations:** Department of Vascular and Interventional Radiology, Singapore General Hospital, 31 Third Hospital Ave, Singapore, 168753 Singapore

**Keywords:** Coaxial, Embolisation, Superselective, Triaxial

## Abstract

**Learning objective:**

To share our experience in utilising the triple coaxial (triaxial) system in superselective cannulation of arteries for complex embolisation procedures.

**Background:**

Percutaneous transcatheter selective embolisation is widely performed for a myriad of oncologic (e.g., trans-arterial chemo- or radio-embolisation) and non-oncologic (e.g., for embolisation of bleeding and benign conditions such as uterine fibroid and benign prostate hyperplasia) purposes.

The cornerstone of such embolisation procedures is to achieve superselective cannulation of the arterial supply to the tumour/organ, preventing the complication of non-target embolisation. However, a multitude of factors, such as complex vascular anatomy, can pose challenges to achieving this goal.

**Clinical findings/procedural details:**

The triaxial system utilises two smaller microcatheters telescoped through each other over a microwire. We have adopted the triaxial system for specific cases due to its perceived superior torquability and trackability compared to the conventional coaxial system, in which superselective cannulation is anticipated to be challenging. The triaxial system is also favourable in situations where the inner microcatheter needs to be “sacrificed” after administering the embolics (e.g., after administering radioisotopes in radioembolisation, N-butyl cyanoacrylate [NBCA] glue or ethylene–vinyl alcohol copolymer [EvOH] Onyx). Through a case series with procedural details such as fluoroscopic time, contrast administered, etc., we hope to illustrate the utility and efficacy of the triaxial system as well as present pitfalls in its usage.

**Conclusion:**

The triaxial system appears to be a valuable system for certain complex embolisation procedures and could be better suited than the conventional coaxial systems in these specific circumstances.

## Introduction

Percutaneous transcatheter embolisation is a well-established and widely performed minimally invasive therapeutic option for a variety of clinical conditions such as transcatheter arterial chemoembolisation or radioembolisation for hepatocellular carcinoma (HCC), bronchial artery embolisation (BAE) for haemoptysis, prostate artery embolisation for benign prostatic hyperplasia (BPH), embolisation for gastrointestinal bleeding, and embolisation of type II endoleaks following endovascular aneurysm repair (EVAR) (Shimohira et al. 2012, 2013 and 2015; Koganemaru et al. 2016). The goal of embolisation is to occlude or reduce blood or lymph flow in the arterial, venous, or lymphatic system.

One of the key cornerstones of embolisation is superselective catheterisation. This involves advancing the catheter tip within the feeding vessel as close as possible to the target lesion with the goal of delivering the payload / embolic agents to achieve effective embolisation and minimise the risk of non-target vessel embolisation, which may result in disastrous consequences. For example, in the case of BAE, superselective cannulation is performed whilst paying particular attention to the microcatheter tip, which is guided beyond the origin of the anterior medullary arteries or the great anterior radiculomedullary artery (artery of Adamkiewicz) to avoid the dreaded complication of spinal cord ischemia/infarction (Tanaka et al. 1997).

Tortuous, narrowed, and complex vascular anatomy can pose a problem in achieving superselective embolisation. Vessel tortuosity can result in a lack of pushability of the microcatheters and the loss of guidewire torque essential for the successful cannulation of a vessel branch. Tortuous vessels may also prevent the all-around transmission of energy over the microcatheter and occasionally leads to sudden forward motion leading to vessel spasm and injury such as a dissection and/or perforation (Cherian 2018). This can result in complications, failure of the procedure, and longer procedure duration with implications for hospitalisation stay and cost.

In recent years, the immense improvement in microcatheters, guidewires, and digital angiographic equipment and technology have enabled more peripheral superselective catheterisation of distal vessels. And with the availability of an even smaller microcatheter recently, we have adopted this new technique, where a smaller inner microcatheter is introduced into a larger intermediate microcatheter parked inside a base catheter called the triple coaxial (triaxial) system to perform superselective catheterisation and embolisation of various conditions.

## Materials and methods

We conducted a retrospective audit of 10 cases at our institute, where the triaxial system was utilised for various complex embolisation cases from 2020 to 2021. Two interventional radiologists (AG and ST) performed the cases with an experience of > 10yrs and > 5yrs, respectively. The recorded procedural details, such as duration of the procedure, amount of contrast media utilised, fluoroscopy time, radiation dose, and procedural success, were captured for audit. The procedures were performed using the Canon Medical Alphenix (Tochigi, Japan) or Siemens Medical Artis (Erlangen, Germany) interventional angiography system. Iohexol (Omnipaque 350, GE Healthcare, Illinois, USA) was used as contrast media. No approval from our institutional review board was necessary for this retrospective anonymised report.

### Devices used in the triaxial system

In our institution, most embolisation procedures are routinely performed via a common femoral or radial artery approach. Various configurations of 4 or 5-Fr. angiographic catheters are employed as the base catheter. The most commonly utilised catheters for femoral access are the Cobra 2 (C2) (Cordis, Florida, USA), Shepherd Hook (SHK) (Cordis, Florida, USA), and the Simmons 1 (SIM1) (Cordis, Florida, USA). Ultimate 1 (ULT1) (Merit Medical, Utah, USA), Tiger (TIG) (Terumo Medical, Tokyo, Japan), and Multipurpose 1 (MPA1) (Cordis, Florida, USA) are commonly used for radial access.

**Fig. 1:**
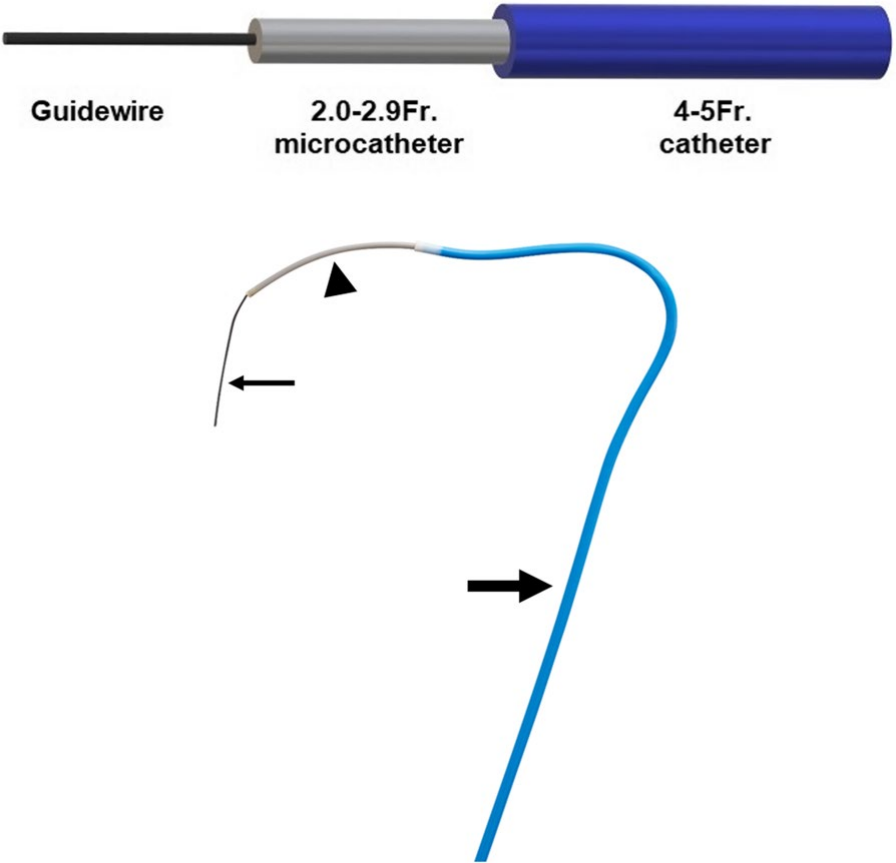
Sketch showing the composition of a conventional coaxial system. It consists of a 0.014 to 0.018-inch microguidewire (small arrow), a microcatheter usually ranging from 2.0 to 2.9-Fr. (arrowhead) inserted via a 4 or 5-Fr. catheter (large arrow)

Conventionally, within the base catheters, microcatheters, usually ranging from 2.0 to 2.9-Fr. are inserted coaxially to achieve superselective catheterisation of the desired vessel (Fig. 1). A Y-connector/Tuohy Borst adapter system between the base and microcatheters are regularly utilised however the use of heparin flushes are left to the discretion of the operators.

**Fig. 2:**
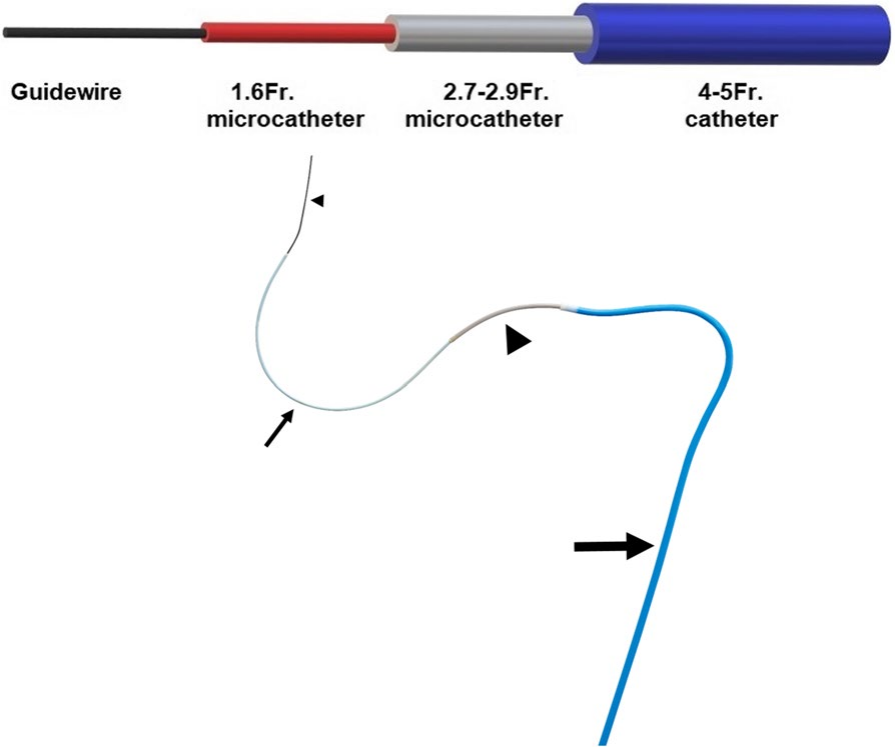
Sketch showing the composition of a triple coaxial system. The triaxial system comprises a 0.014-inch microguidewire (small arrowhead), 1.6-Fr. microcatheter (small arrow), a 2.7 to 2.9-Fr. intermediate microcatheter (large arrowhead), a 4 to 5-Fr. catheter (large arrow)

The triaxial system was used in specific situations where intricate vascular anatomy was anticipated or when the conventional coaxial system was unsuccessful in achieving superselective cannulation. A small inner microcatheter (CARNELIAN, 1.6-Fr., 135 cm shaft length, Tokai Medical Products Inc, Kasugai, Japan) is inserted into a larger intermediate microcatheter (CARNELIAN, 2.7-Fr., 125 cm shaft length, Tokai Medical Products Inc, Kasugai, Japan or PROGREAT, 2.7-Fr., 110 cm shaft length, Terumo Medical, Tokyo, Japan) over a microguidewire (TRANSEND EX 0.014 inch, 200 cm length, Boston Scientific, Massachusetts, USA) within the 4 or 5 Fr. base catheter (Fig. 2). The larger 2.7-Fr. microcatheter allows higher quality angiograms as a high rate and volume of contrast media can be injected via these catheters. It also enables the deployment of larger coils and particulate embolics. The smaller 1.6 Fr. microcatheter is softer and more flexible, allowing cannulation of arteries with more complex anatomy.

## Results

All 10 procedures were completed with successful embolisation of the target vessel(s). Transfemoral approach was used in nine cases with one transradial approach. Six of 10 cases were hepatic artery embolisation due to the relatively high number of hepatic artery interventions like transarterial chemoembolisation (TACE) and transarterial radioembolisation (TARE) being performed in our institute. The decision to use the triaxial system was based on the operator's experience in anticipation of difficulty in negotiating the target vessels upon reviewing the preoperative CT angiogram or the initial catheter angiogram performed via the base catheter. A range of procedures was performed depending on the location, with an assorted range of parameters obtained (summarised in Table 1).

**Table 1:** Procedural parameters for the 10 patients who underwent various embolisation procedures using the triaxial system

Procedure	Embolic used	Procedure time (min)	Contrast media usage (ml)	Fluoroscopy time (min)	DAP (Gy.cm^2^)	Reference Fluoroscopy time (min)			Reference DAP (Gy.cm^2^)		
						Mean	Min	Max	Mean	Min	Max
BAE	PVA (250 – 355 microns)	129	140	16.3	84.79	25.7	10.9	46.5	138.20	72.20	314.53
Endoleak embolisation (intercoastal artery)	NBCA glue with Lipiodol (1:3 ratio) and detachable coils	160	55	35.5	190.56	29.8	14.3	44.5	140.97	109.72	302.54
Endoleak embolisation (Iliolumbar artery)	NBCA glue with Lipiodol (1:3 ratio)	182	125	39.6	286.41	29.8	14.3	44.5	140.97	109.72	302.54
PAE	Embosphere microspheres (100–300 microns)	130	75	19.5	224.58	30.9	15.5	48.3	450.70	248.30	791.73
TACE	Doxorubicin- Lipiodol emulsion (1:2 ratio)	134	140	14.0	171.36	14.8	2.7	48.7	270.12	20.46	615.74
TACE	Doxorubicin- Lipiodol emulsion (1:2 ratio)	165	95	18.6	213.10	14.8	2.7	48.7	270.12	20.46	615.74
TACE	Doxorubicin- Lipiodol emulsion (1:2 ratio)	109	50	22.3	239.02	14.8	2.7	48.7	270.12	20.46	615.74
TACE	Doxorubicin- Lipiodol emulsion (1:2 ratio)	198	76	28.0	281.67	14.8	2.7	48.7	270.12	20.46	615.74
MAA^a^	Macro-Agglutinated Albumin Technetium 99 m	138	87	10.6	121.11	14.8	2.7	48.7	270.12	20.46	615.74
Y-90^a^	Resin microspheres (SIRTex)	192	95	25.6	271.45	14.8	2.7	48.7	270.12	20.46	615.74

^a^Same reference range for fluoroscopy time and DAP were used for TACE and TARE due to the technical similarities between the two procedures

### Clinical applications

#### Transarterial chemoembolisation (TACE) for hepatocellular carcinoma (HCC)

TACE is commonly utilised to treat patients with Barcelona Clinic Liver Cancer (BCLC) intermediate stage (B) HCC who are not eligible for curative surgery or percutaneous ablation.

**Fig. 3:**
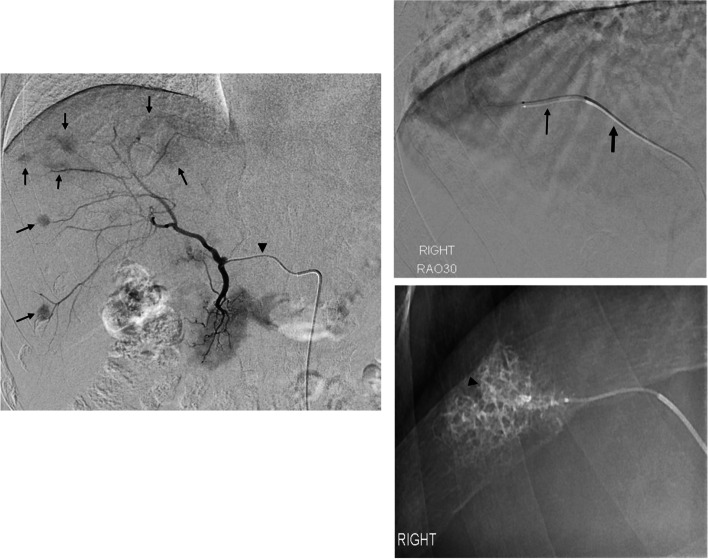
**a** Digital subtraction angiography (DSA) performed via 2.7-Fr. high-flow microcatheter (arrowhead) in a 78-year-old man showing multifocal HCCs (arrows) in the right lobe of the liver. **b** Superselective TACE was performed for the tumor in segment VII from a distal branch of a subsegmental artery using the triaxial system (small arrow—1.6-Fr. microcatheter; large arrow—2.7-Fr. intermediate microcatheter). **c** Post-TACE spot radiograph obtained showing lipiodol staining of the tumour (arrowhead) and the adjacent portal veins in the embolised region

Ultraselective TACE is preferable to nonselective TACE because a selective approach increases the effectiveness of treatment on tumours while reducing the damage and toxicity to the adjacent normal tumour-free liver (Miyayama et al. 2007). Therefore, manoeuvring and advancing the microcatheter tip within the feeding artery as close as possible to the tumour is an indispensable step in performing ultraselective TACE, albeit sometimes difficult due to the inherent tortuosity of these arteries commonly found in cirrhotic livers (Fig. 3). Particularly in tortuous vascular anatomy, the progression of a microcatheter over the guidewire (conventional coaxial technique) may be difficult or impossible due to the retrograde kickback (bascule) of the guidewire and/or the microcatheter outside the target vessel. Therefore, using a triaxial platform in performing ultra-selective TACE would be more useful (Shimohira et al. 2011 and 2012) for achieving higher local control rates for HCC.

#### Transarterial radioembolisation (TARE) for hepatocellular carcinoma (HCC)

**Fig. 4:**
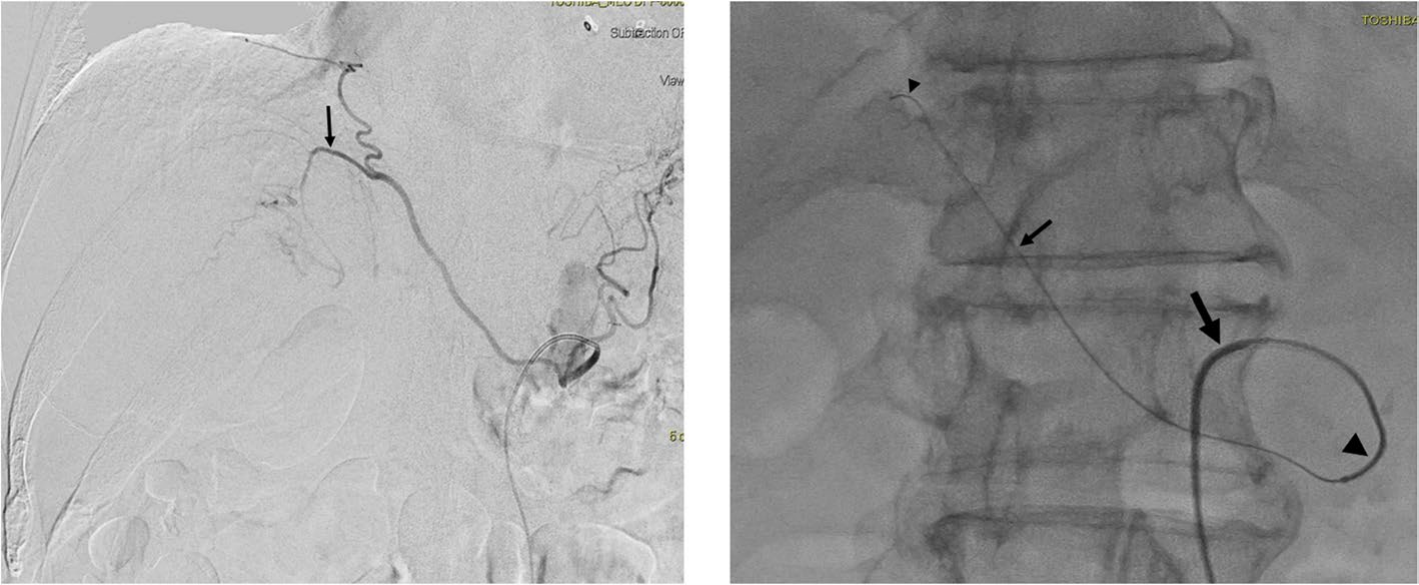
**a** DSA of the right inferior phrenic artery (RIPA) arising from the left gastric artery in a 74-year-old woman with a large HCC in the right lobe of the liver. The patient is planned for radioembolisation with yttrium-90 (Y-90). As noted from this angiogram, the HCC is noted to be partly supplied by the posterior (inferior) branch of the RIPA (arrow). **b** Superselective catheterization of the posterior branch of the RIPA performed using a triaxial system (small arrowhead—0.014-inch microguidewire; small arrow—1.6-Fr. microcatheter; large arrowhead—2.7-Fr. intermediate microcatheter; large arrow—4-Fr. catheter). Coil embolisation of the anterior (superior) branch of the RIPA was performed, followed by delivery of the Y-90 microspheres into the posterior branch (not shown)

Transarterial radioembolisation with yttrium-90 (Y-90) microspheres is an established treatment for unresectable or advanced HCC where the patient has a large tumour burden or lobar portal vein thrombosis. When radioembolisation is performed using a lobar approach, patients may be at risk of hepatotoxicity, as most of these patients have some degree of underlying existing hepatic dysfunction. Like the concept of ultraselective TACE, superselective radioembolisation can reduce the damage and toxicity to the adjacent normal tumour-free hepatic parenchyma, and a higher tumouricidal dose can be safely delivered, potentially yielding better response rates without compromising the patient’s safety (Padia et el. 2014). Most, if not all, of these patients, have cirrhotic livers with inherently tortuous arteries. With the growing demand for superselective catheterisation during TARE, an interventionist can deploy the triaxial system when faced with such challenging anatomy (Fig. 4). For TARE, the triaxial catheter system also confers the advantage of being able to discard the inner smaller microcatheter after administering the radioisotope to prevent contamination.

#### Bronchial artery embolisation (BAE) for haemoptysis

BAE is a well-established interventional procedure for managing haemoptysis with an immediate clinical success rate in controlling haemoptysis up to 90% (Yoon et al. 2002).

**Fig. 5:**
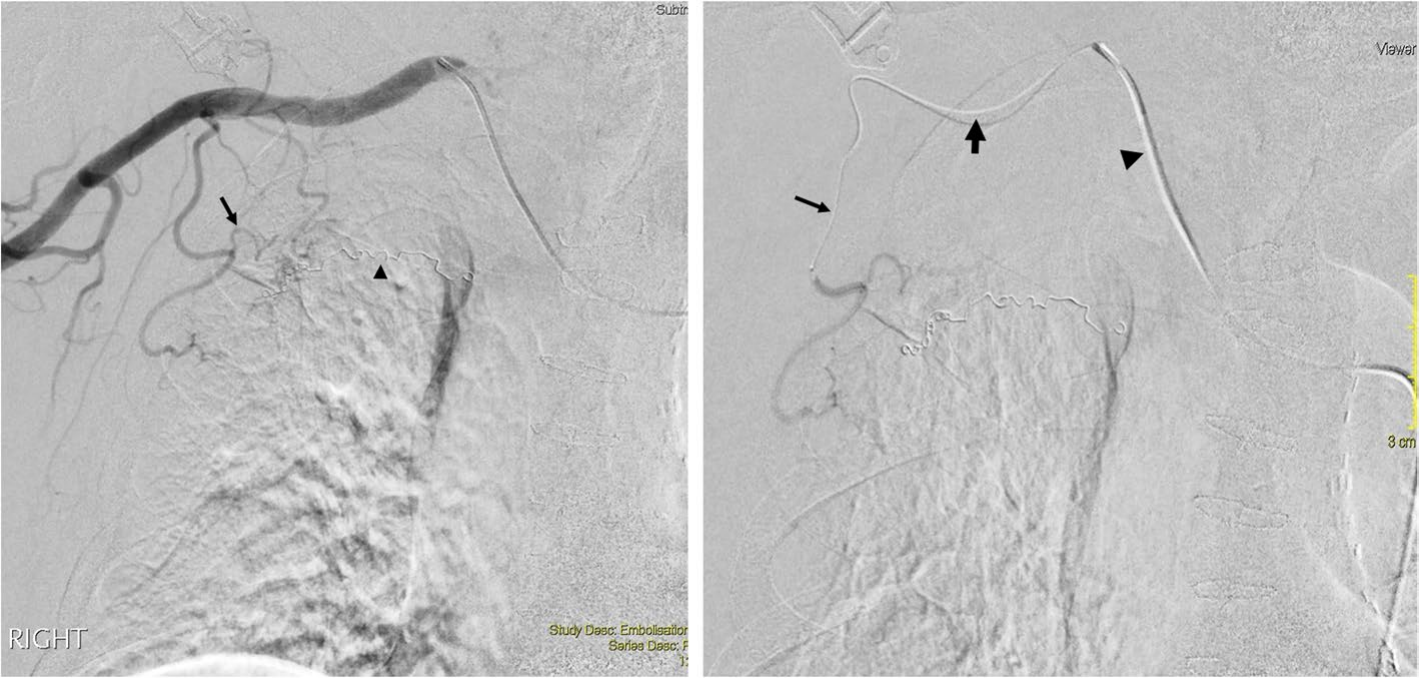
**a** DSA of the right subclavian artery in a 72-year-old man presenting with massive haemoptysis due to prior pulmonary tuberculosis. Abnormal supply to the right upper lobe is noted from the lateral thoracic artery (arrow). Note the embolisation coils (arrowhead) deployed prior to this angiogram in the right intercoastal bronchial trunk due to bronchial artery-pulmonary vein shunting. **b** Successful selective catheterisation of the offending branch was performed using a triaxial system (small arrow—1.6-Fr. microcatheter; large arrow—2.7-Fr. intermediate microcatheter; arrowhead – 4-Fr. catheter) after multiple attempts to cannulate this branch with a conventional coaxial system failed

One of the most severe and dreaded complications is inadvertent embolisation of a spinal artery leading to spinal cord ischaemia and paralysis. The incidence of spinal artery ischaemia from BAE is between 0.6% and 4.4% (Panda et el. 2017). Hence superselective catheterisation distal to the spinal artery feeder is critical in BAE to minimise the risk of spinal cord ischaemia. Conversely, vessel tortuosity is a common angiographic finding with a pathological bronchial artery or non-bronchial systemic arteries (Kalva 2009), creating a potential obstacle for navigating the microcatheter for superselective catheterisation (Fig. 5). Woo et al. 2013 reported technical failures in 17 out of 293 cases (5.8%), of which the following difficulties were associated with the target artery: tortuosity of the pathological artery in 13 cases, orifice stenosis in two cases, a small calibre in one case and an acute angle of branching in one case. A triaxial system can potentially overcome these technical challenges.

Studies have also shown that BAE performed with n-butyl cyanoacrylate (NBCA) / glue provides a higher haemoptysis-free survival rate than the more commonly used polyvinyl alcohol (PVA) particles without increasing complication rates in patients with bronchiectasis due to its more durable embolic effect than PVA particles. However, NBCA is known for its difficulties when handling as the catheter tip may adhere to the vessel wall due to its rapid rate of hardening (polymerisation). Hence the microcatheter needs to be rapidly withdrawn after embolisation to prevent this issue. With the triaxial system, access to the feeding artery is still maintained with an intermediate 2.7-Fr. microcatheter, so even if the first embolisation attempt was inadequate, the smaller inner microcatheter can easily be introduced again into the target vessel without losing access.

Additionally, NBCA casts sometimes adhere to the microcatheter tip when removing the microcatheter. In the conventional coaxial system, this situation may result in migration of the cast to the aorta or the spinal artery leading to non-target embolisation and potentially serious complications. However, with the triaxial system, the intermediate 2.7-Fr. microcatheter can scrape the cast off the smaller microcatheter within the bronchial artery, and the cast can then be carried or flushed away to a safe distal site (Shimohira et al. 2015).

#### Embolisation of type II endoleak after Endovascular Aneurysm Repair (EVAR)

After EVAR, transcatheter arterial embolisation is a standard treatment option for persistent type II endoleak. (Jones et al. 2007) reported late aortic rupture in about 6% of patients with persistent type II endoleak, with or without associated aneurysm enlargement.

**Fig. 6:**
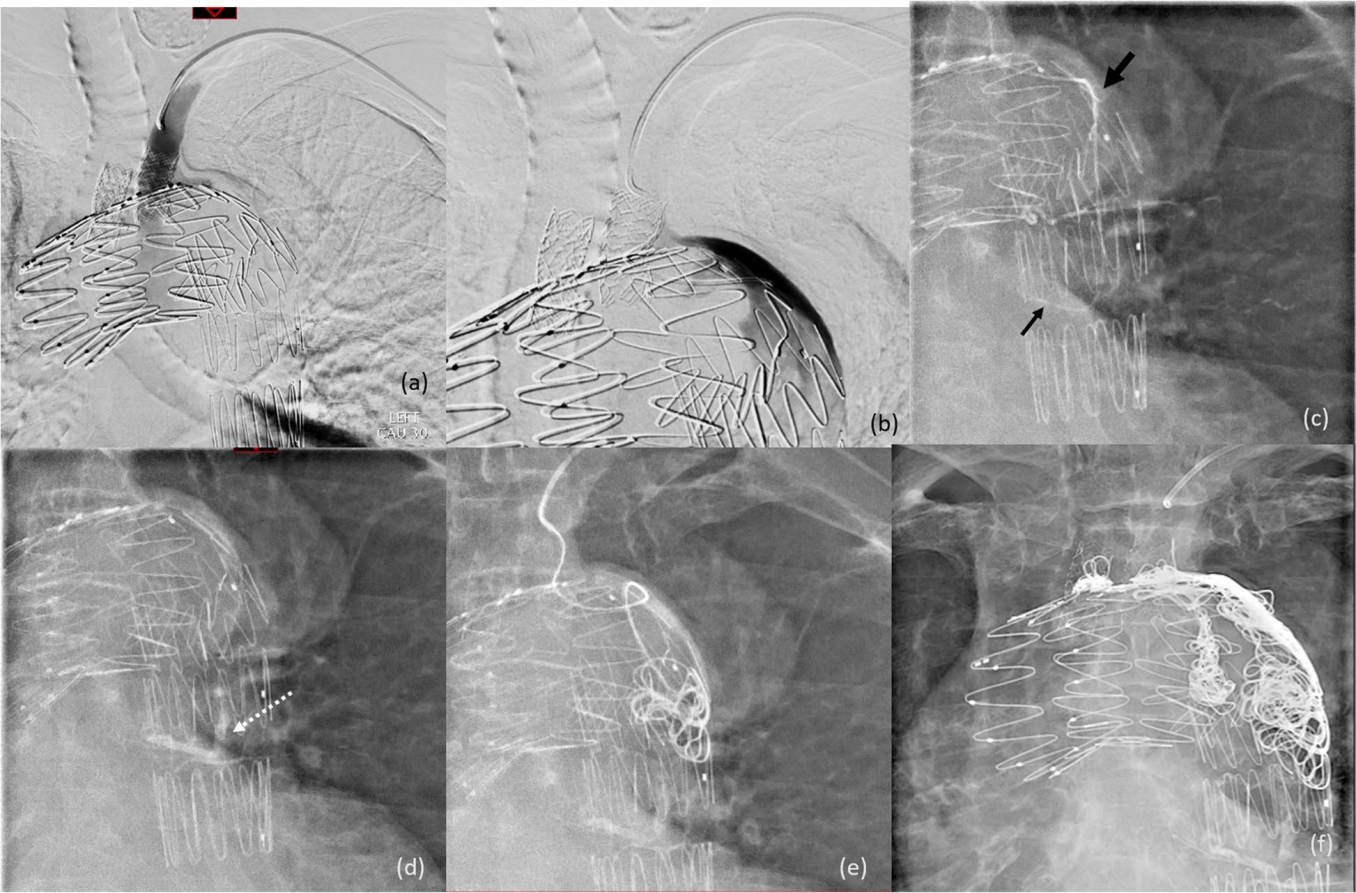
**a** and **b** DSAs from the left subclavian artery demonstrating a Type II endoleak post thoracic endovascular aortic repair (TEVAR) with opacification of the aneurysm sac from one of the left intercostal artery. **c** Selective cannulation of the efferent left intercostal artery with the 1.6-Fr. microcatheter (small arrow) with the stability offered by the 2.7-Fr. intermediate microcatheter (large arrow). This artery was embolised with NBCA glue (1:3 dilution with lipiodol). **d** Glue cast within the embolised intercostal artery. **e** The 1.6-Fr. microcatheter was discarded, and the rest of the sac was embolised with multiple Ruby detachable coils (Penumbra, California, USA) via the intermediate 2.7-Fr. microcatheter. **f** Completion image showing exclusion of the aneurysm sac

Type II endoleak is the most common type of endoleak. It is related to the retrograde flow via collaterals arteries, namely the inferior mesenteric artery (IMA), intercostal, lumbar arteries, and branches of the internal iliac artery. These collaterals can be tortuous and long, making it technically challenging to perform selective catheterisation and embolisation retrogradely from the parent vessel. One solution to this is the Squeeze Technique described by Kang et al., for which the culprit artery is cannulated via the small triangular space between the stent graft and the aorta, otherwise described as the “free graft skirt” (Kang et el. 2014). The issue with the Squeeze Technique is the difficulty in cannulating the efferent small target artery, which takes off a large space (i.e., the aneurysm sac). With the triaxial system, the larger intermediate 2.7-Fr. microcatheter supports and provides increased stability to the more flexible smaller inner microcatheter to increase the chance of successful cannulation of the target vessel with the help of a shapable tip microguidewire. This use is illustrated in the case shown in Fig. 6.

Depending on the operator’s choice, liquid embolic agents such as NBCA / glue or Onyx (ethylene–vinyl alcohol [EVOH] copolymer with dimethyl sulfoxide [DMSO] solvent and micronized tantalum powder) are occasionally used for the embolisation of the type II endoleaks requiring multiple doses. Access to the feeding artery can still be maintained via the intermediate 2.7-Fr. microcatheter, even if the inner microcatheter has to be replaced, saving on procedure time and radiation (Shimohira et al. 2013).

### Prostate artery embolisation (PAE) for benign prostatic hyperplasia (BPH)

Prostatic arterial embolisation (PAE) is a safe and effective treatment option for lower urinary tract symptoms caused by BPH. It has a high technical success rate with improved urinary flow rates and quality of life (Pisco et al. 2016). The "PErFecTED" (Proximal Embolisation First, Then Embolise Distal) technique described by (Carnevale FC et al. 2014), where the prostate artery is embolised proximally first, and then the microcatheter is advanced distally into the intraprostatic parenchyma branches for embolisation has shown more significant prostatic ischaemia and infarction with clinical improvement of lower urinary symptoms and lower recurrence rates (Carnevale et al. 2014).

However, PAE can be technically challenging, especially given that the general age group of these patients is more advanced with concurrent medical conditions leading to tortuous arterial anatomy. Additionally, the prostatic arteries often stem at an acute angle and exhibit atherosclerotic narrowing. Moreover, the degree of atherosclerotic narrowing is significantly correlated with the BPH symptoms, such that the most symptomatic patients may have the most challenging arterial anatomy (Haga et al. 2018). When faced with a tortuous and narrowed prostatic artery with angulated origins, the triaxial system may prove more advantageous than the conventional system, given its greater ability to negotiate such anatomy to attain the PErFecTED technique.

## Discussion

Anecdotal observations in interventional radiology suggest that complex vascular anatomy compounded by increased tortuosity and luminal narrowing correlates with extended procedure time, which translates to longer fluoroscopy time (higher radiation doses) and greater demand for contrast agents with higher risks of technical failure.

A microcatheter catheter system that provides better stability, torquability, and trackability is pivotal to increasing the technical success rate in endovascular interventions. With the triaxial system, the 2.7-Fr. microcatheter as an intermediate catheter provides more stability in the system's position and prevents the microcatheter's springing forward or sagging when faced with a tortuous and challenging vascular anatomy.

Our experiences with these cases demonstrate that the triaxial system could have benefits over the conventional coaxial system in the aforementioned described situations. Besides providing a more stable platform for selective embolisation, we can avoid spasms and vascular injury due to repetitive manipulation of the microcatheter resulting in failure of the catheterisation (or procedure). This system also provides certain advantages when liquid embolic agents such as NBCA or Onyx are utilized, as in BAE and the embolisation of type II endoleaks. Especially in the case of NBCA, due to its faster polymerisation rate, the operator needs to quickly withdraw the microcatheter after injection to prevent tip adherence to the vessel wall, with the potential of losing hard-earned vascular access. With this system, access to the feeding artery can still be maintained, and a smaller microcatheter can conveniently be re-introduced into the target vessel without losing access and time should the embolisation be inadequate in the first attempt. Additionally, the larger 2.7-Fr microcatheter can serve another function of scraping off any adhered NBCA or Onyx casts on the smaller microcatheter's tip when withdrawing, which can then be flushed away to a safe site.

Similarly, the other benefit of this system is that vascular access is still maintained with the 2.7-Fr. intermediate microcatheter in situations where the smaller microcatheter needs to be discarded, either due to its luminal blockage due to an NBCA cast or after delivery of the payload, in case of TARE, where more than one injection is required. To avoid contamination, discarding the microcatheter utilised to deliver the radioisotope is a good practice due to the residual activity (Robert 2020). This action is coined the "pump-and-dump" technique by the authors.

All the procedures using the triaxial system were performed within acceptable parameters in the current literature, especially from a radiation point of view. The means of fluoroscopy time and dose area product (DAP) of patients undergoing TACE in the current literature ranges from 2.7—48.7 min and 20.46—615.74 Gy.cm^2^, respectively (Miller et al. 2003), for BAE ranges from 10.9—46.5 min and 72.20—314.53 Gy.cm^2^ (Robert 2020), for embolisation of Type II endoleak ranges from 14.3—44.5 min and 109.72—302.54 Gy.cm^2^ (Ierardi et al. 2018) and for PAE ranged from 15.5—48.3 min and 248.3—791.73 Gy.cm^2^ (Andrade et al. 2017).

Besides the specific cases mentioned above, the triaxial system can also be utilised for embolisation of lower gastrointestinal bleeding (Shimohira et al. 2015) and re-embolisation of recanalised pulmonary arteriovenous malformations (Shimohira et al. 2015).

Although the triaxial system has an advantage over the conventional coaxial system when using liquid embolic agents such as NBCA and Onyx. Given the smaller inner diameter of the smaller microcatheter, there are limitations to the size of particulate embolic agents such as PVA particles, microspheres, beads, and coils that can be deployed. The system can accommodate particles smaller than < 300 μm, smaller gelatin sponge particles, and 0.014-inch microcoils. Larger particulate embolic agents may result in the occlusion of the microcatheter. Therefore, the recommended size of embolic agents must be available in the inventory before using the triaxial system.

Another drawback would be the higher cost due to an additional microcatheter’s usage, adding approximately 200 USD more locally. Nevertheless, when faced with challenging vascular anatomy with an extended access route, the triaxial system could prove to be advantageous. We also believe the additional cost is justified, as it shortens the procedure time, which translates to lesser radiation exposure and possibly lower risk of more adverse events.

## Conclusion

The triaxial system appears to be a valuable system that can be utilised for a myriad of conditions, mainly when complex embolisation procedures are performed. Nevertheless, additional studies with a larger cohort are required to further substantiate this technique's usefulness.

## Data Availability

Data and material used in this manuscript can be made available on request.

## Abbreviations

BCLC: Barcelona Clinic Liver Cancer
BPH: Benign prostatic hyperplasia
BAE: Bronchial artery embolisation
DSA: Digital subtraction angiography
DMSO: Dimethyl-sulfoxide
EVAR: Endovascular Aneurysm Repair
EVOH: Ethylene–vinyl alcohol
HCC: Hepatocellular carcinoma
NBCA glue: N-butyl cyanoacrylate
PVA: Polyvinyl alcohol
PAE: Prostate artery embolisation
RIPA: Right inferior phrenic artery
TEVAR: Thoracic endovascular aortic repair
TACE: Transarterial chemoembolisation
TARE: Transarterial radioembolisation
Y-9: 0Yttrium-90

## References

[CR1] Andrade G, Khoury HJ, Garzón WJ, Dubourcq F, Bredow MF, Monsignore LM, Abud DG (2017). Radiation Exposure of Patients and Interventional Radiologists during Prostatic Artery Embolization: A Prospective Single-Operator Study. J Vasc Interv Radiol.

[CR2] Carnevale FC, Moreira AM, Antunes AA (2014). The "PErFecTED technique": proximal embolization first, then embolize distal for benign prostatic hyperplasia. Cardiovasc Intervent Radiol.

[CR3] Cherian M, Mehta P, Santhosh P, Rahul KR, Jenny G, Elango S (2018). Overcoming Tortuous Anatomy in Intracranial Intervention. J Clin Interv Radiol ISVIR.

[CR4] Drescher R (2020). Transarterial Radioembolization with Yttrium-90 Glass Microspheres: Distribution of Residual Activity and Flow Dynamics during Administration. J Vasc Interv Radiol.

[CR5] Haga N, Akaihata H, Hata J (2018). The association between local atherosclerosis of the prostatic artery and benign prostatic enlargement in humans: Putative mechanism of chronic ischemia for prostatic enlargement. Prostate.

[CR6] Ierardi AM, Pesapane F, Rivolta N, Fumarola EM, Angileri SA, Piacentino F, Carrafiello G (2018). Type 2 endoleaks in endovascular aortic repair: cone beam CT and automatic vessel detection to guide the embolization. Acta Radiol.

[CR7] Jones JE, Atkins MD, Brewster DC (2007). Persistent type 2 endoleak after endovascular repair of abdominal aortic aneurysm is associated with adverse late outcomes. J Vasc Surg.

[CR8] Kalva SP (2009). Bronchial artery embolization. Tech Vasc Interv Radiol.

[CR9] Kang HJ, Kim CW, Lee TH (2014). Endovascular Treatment of Type II Endoleak Following Thoracic Endovascular Aortic Repair for Thoracic Aortic Aneurysm: Case Report of Squeeze Technique to Reach the Aneurysmal Sac. J Korean Soc Radiol.

[CR10] Koganemaru M, Nonoshita M, Iwamoto R, Kuhara A, Nabeta M, Kusumoto M, Kugiyama T, Nagata S (2016). Abe T Ultraselective embolization using a 1.7-Fr catheter and soft bare coil for small intestinal bleeding. Minim Invasive Ther Allied Technol.

[CR11] Miller DL, Balter S, Cole PE, Lu HT, Berenstein A, Albert R, Schueler BA, Georgia JD, Noonan PT, Russell EJ, Malisch TW, Vogelzang RL, Geisinger M, Cardella JF, George JS, Miller GL, Anderson J (2003). Radiation doses in interventional radiology procedures: the RAD-IR study: part II: skin dose. J Vasc Interv Radiol.

[CR12] Miyayama S, Matsui O, Yamashiro M (2007). Ultraselective transcatheter arterial chemoembolization with a 2-f tip microcatheter for small hepatocellular carcinomas: relationship between local tumor recurrence and visualization of the portal vein with iodized oil. J Vasc Interv Radiol.

[CR13] Padia SA, Kwan SW, Roudsari B, Monsky WL, Coveler A, Harris WP (2014). Superselective yttrium-90 radioembolization for hepatocellular carcinoma yields high response rates with minimal toxicity. J Vasc Interv Radiol.

[CR14] Panda A, Bhalla A, Goyal A (2017). Bronchial artery embolization in hemoptysis: a systematic review. Diagn Interv Radiol.

[CR15] Pisco JM, Bilhim T, Pinheiro LC (2016). Medium- and long-term outcome of prostate artery embolization for patients with benign prostatic hyperplasia: results in 630 patients. J Vasc Interv Radiol.

[CR16] Shimohira M, Ogino H, Kawai T (2011). use of the triaxial microcatheter method in super-selective transcatheter arterial chemoembolisation for hepatocellular carcinoma. Br J Radiol.

[CR17] Shimohira M, Ogino H, Kawai T (2012). Clinical usefulness of the triaxial system in super-selective transcatheter arterial chemoembolization for hepatocellular carcinoma. Acta Radiol.

[CR18] Shimohira M, Hashizume T, Suzuki Y, Kurosaka K, Muto M, Kitase M, Mizutani M, Shibamoto Y (2013). Triaxial system for embolization of type II endoleak after endovascular aneurysm repair. J Endovasc Ther.

[CR19] Shimohira M, Hashizume T, Kawai T, Muto M, Ohta K, Suzuki K, Shibamoto Y (2015). Triaxial system in re-embolization for recanalization of pulmonary arteriovenous malformations. Pol J Radiol.

[CR20] Shimohira M, Hashimoto T, Abematsu S, Hashizume T, Nakagawa M, Ozawa Y, Sakurai K, Shibamoto Y (2015). Triaxial system in bronchial arterial embolization for haemoptysis using N-butyl-2-cyanoacrylate. Br J Radiol.

[CR21] Shimohira M, Hashizume T, Ohta K, Honda J, Shibamoto Y (2015). Triaxial transarterial embolization for lower gastrointestinal bleeding: a retrospective case series. Minim Invasive Ther Allied Technol.

[CR22] Tanaka N, Yamakado K, Murashima S (1997). Superselective bronchial artery embolization for hemoptysis with a coaxial microcatheter system. J Vasc Interv Radiol.

[CR23] Woo S, Yoon CJ, Chung JW, Kang SG, Jae HJ, Kim HC (2013). Bronchial artery embolization to control hemoptysis: comparison of N butyl- 2-cyanoacrylate and polyvinyl alcohol particles. Radiology.

[CR24] Yoon W, Kim JK, Kim YH (2002). Bronchial and nonbronchial systemic artery embolization for life threatening hemoptysis: A comprehensive review. Radiographics.

